# Platelet-Rich Plasma Injections as an Alternative to Surgery in Treating Patients With Medial Epicondylitis: A Systematic Review

**DOI:** 10.7759/cureus.28378

**Published:** 2022-08-25

**Authors:** Wael M Alzahrani

**Affiliations:** 1 Department of Surgery, College of Medicine, Najran University, Najran, SAU

**Keywords:** golfer’s elbow, platelet-rich plasma, epicondylitis, elbow pain, medial epicondylitis

## Abstract

To synthesize the available information on the effectiveness of platelet-rich plasma (PRP) injections against surgery as therapy strategies for medial epicondylitis (ME). We searched the Embase, MEDLINE, and Cochrane Library databases with the relevant keywords to identify the studies comparing the efficiency of PRP injections and ME surgery. We excluded non-English articles, case reports, and conference abstracts. Only two studies met the inclusion criteria and were included in the qualitative synthesis. No conflicts were reported between both studies. Both studies were carried out in the United States of America. The outcomes of PRP and surgical interventions were similar, with no reported statistical differences. Both studies recorded an excellent outcome following the PRP and surgical interventions, where the patients returned to full movement with no pain. The current evidence shows that PRP injections are just as effective as ME surgery in relieving pain and restoring function for those with ME, especially in the short and mid-term. Therefore, the injection of PRP is a promising treatment option for ME.

## Introduction and background

Medial epicondylitis (ME), sometimes known as "golfer's elbow," is a condition commonly seen by orthopedic surgeons [1]. Although the prevalence of ME is low (1%), between 3.8% and 8.2% of people may experience ME during work [2,3]. Extracorporeal shock wave therapy (ESWT) and modified surgical procedures have been proposed by many studies, despite the fact that non-surgical therapies remain cornerstones of ME treatment [4]. Patients who have no symptoms related to the ulnar nerve are classified as having type 1 ME. The severity of ulnar nerve involvement is used to classify type 2 ME. Type 2A presents with ulnar nerve symptoms but no objective deficit on physical exam or electromyography, while type 2B presents with objective deficits on both [5]. Differentiating between type 1 and type 2 ME is critical. In contrast to type 2, which needs surgical intervention, the prognosis of type 1 is better since it does not include the ulnar nerve. The outcome of nonoperative treatment of type 2 is worse [5].

The common flexor tendon (CFT) origin is attached to the medial epicondyle and is composed of the flexor carpi ulnaris (FCU), flexor carpi radialis (FCR), flexor digitorum superficialis (FDS), pronator teres (PT), and palmaris longus (PL) muscles. In addition to their role as flexors and pronators, these muscles also help maintain the dynamic stability of the elbow joint [6]. The extreme valgus force produced by the elbow causes acute microtrauma in the flexor-pronator group connected to the medial epicondyle [7]. Nevertheless, the pronator teres and the flexor carpi radialis are the most likely muscles in this group to be impacted [8]. Patients often complain of medial elbow pain, which is made worse by activities such as wrist flexion or resistant forearm pronation [9].

More than 90% of type 1 patients with ME report considerable symptom improvement after receiving non-surgical treatments [10]. Corticosteroid injections, anti-inflammatory medicines, physical therapy, bracing, and activity adjustment are some of these non-surgical treatment options that may help these patients [8]. Surgery may be the next option if non-surgical therapy has failed after six months [11]. Open debridement and CFT release or repair is the standard surgical treatment for ME; however, Vinod A and Ross G proposed a percutaneous and arthroscopic method to diagnose and repair refractory ME [8].

According to recent studies, platelet-rich plasma (PRP) injections have gained popularity as therapy for musculoskeletal injuries. Plasmapheresis-modified autologous blood is the source of PRP, a solution with massively increased platelet content. This platelet solution has an exceptionally high concentration of many biological components, growth factors, and proteins necessary for soft tissue healing. PRP injections have been proposed to treat various diseases and musculoskeletal injuries [12]. This includes hamstring injuries, osteoarthritis, rotator cuff tears, and a variety of tendinopathies such as lateral epicondylitis (LE). Large-scale and long-term trials are still required to prove this, although early data for using PRP to treat LE is encouraging [13-16]. PRP injections were used effectively to treat cases that failed non-surgical treatment of LE [17], and preliminary data show that they are superior to steroid injections in the short-term management of this condition [18]. Our purpose was to synthesize the available evidence on the efficacy of PRP injections against surgery as therapy strategies for ME. It is hypothesized that PRP injections would provide results comparable to ME surgery.

## Review

Methods

Search Strategy

The Preferred Reporting Items for Systematic Reviews and Meta-Analyses (PRISMA) standards were followed, when applicable, during the reporting of the current systematic review. The protocol of this review was registered with the Open Science Framework. Covering the studies conducted between January 1, 2010, and December 31, 2020, the author searched MEDLINE via the PubMed, Embase, and Cochrane Library databases using the terms “medial elbow pain, elbow tendinopathy, golfer’s elbow, medial epicondylitis, medial epicondylitis surgery, platelet-rich plasma, and platelet-rich plasma injection”. In addition, the reference lists of the prospective eligible articles were further checked to see if there were any close results.

Study Selection and Inclusion Criteria

The inclusion criteria for this study included studies that compared PRP injections with medical surgeries for treating ME (of both type 1 and 2). Besides, the review included only studies written in English. The study excluded all case series, case reports, book chapters, editorials, letters, expert opinions, systematic reviews, and meta-analyses. Title and abstracts of the retrieved studies were screened. The full texts of the articles that fulfilled the inclusion criteria were then obtained.

Data Extraction and Quality Assessment

Data were extracted from the included studies. The characteristics of the included studies, patients, and received interventions were extracted. Moreover, both primary and secondary endpoints were extracted. The quality of included studies was assessed using the Methodological Index for Nonrandomized Studies (MINORS) scale, where 0 = not reported, 1 = inadequately reported, and 2 = adequately reported [19]. The ideal score for the comparative studies is 24 while the ideal score for the noncomparative is 16.

Statistical Analysis

Due to the small number and heterogeneity of studies, a systematic review rather than a meta-analysis was conducted.

Results

Characteristics of the Study

**Figure 1:**
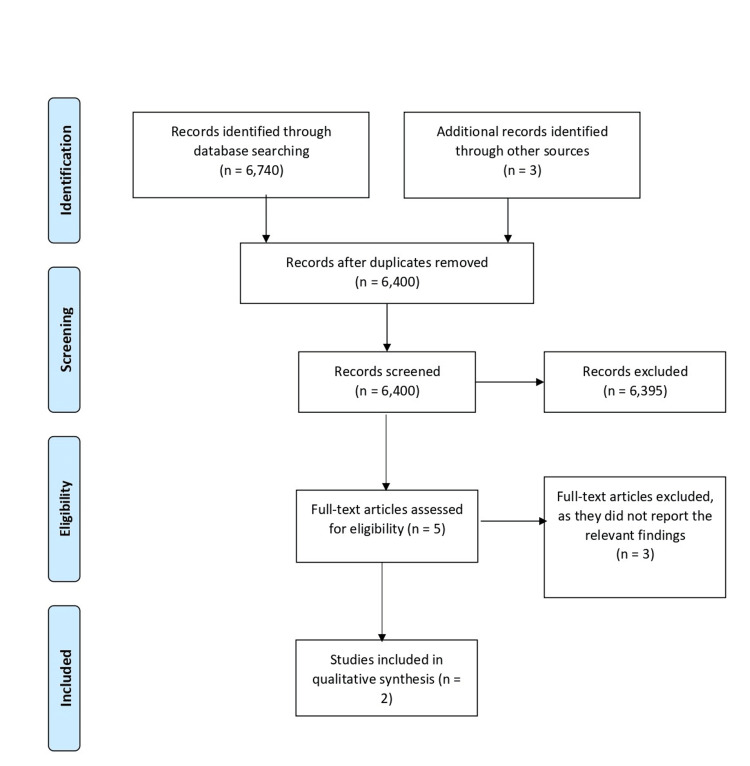
PRISMA flow diagram PRISMA: Preferred Reporting Items for Systematic Reviews and Meta-Analyses

Figure 1 summarizes the search results and publication selection process. The literature search retrieved a total of 6,743 articles. Title and abstract screening resulted in the exclusion of 6,395 articles, and two articles were subjected to full-text screening. The five articles were reviewed, and three were excluded, as they did not directly compare the injection of PRP with the ME surgery. Thus, only two studies were included in the qualitative synthesis [20,21]. The level of evidence for both studies was III. No conflicts were reported in both studies, and both studies were conducted in the United States of America.

Table 1 shows the characteristics of both studies.

**Table 1:** Characteristics of included studies NR: not reported; BMI: body mass index; COI: conflict of interest; PRP: platelet-rich plasma

Characteristics	Bohlen et al. [20]		Boden et al. [21]	
Journal	The Orthopaedic Journal of Sports Medicine		Journal of Shoulder and Elbow Surgery	
Publication year	2020		2019	
Patient enrollment years	2006-2016		2014-2017	
Level of evidence	III		III	
Study design	Cohort study		Retrospective cohort	
Country of study	USA		USA	
Procedures	PRP	Surgery	PRP	Surgery
Sample size	15	18	32	30
Mean age - years	37.5	47.1	47	51
Sex				
Male	12	12	22	18
Female	3	6	10	12
BMI	NR	NR	NR	NR
Dominant Side				
Right	NR	NR	22	17
Left	NR	NR	10	13

Table 2 presents the results of the quality assessment of included studies. In the current investigation, both included studies scored 18 [16,17].

**Table 2:** MINORS Analysis NR: not reported; MINORS: Methodological Index for Nonrandomized Studies

Study	A Clearly Stated Aim	Inclusion of Consecutive Patients	Prospective Collection of Data	End Points Appropriate to the Aim of the Study	Unbiased Assessment of the Study End Point	Follow-Up Period Appropriate to the Aim of the Study	Loss to Follow-Up Less Than 5%	Prospective Calculation of the Study Size	An Adequate Control Group	Contemporary Groups	Baseline Equivalence of Groups	Adequate Statistical Analyses	Total
Bohlen et al. [20]	2	2	2	2	NR	2	2	NR	NR	2	2	2	18
Boden et al. [21]	2	2	2	2	NR	2	2	NR	NR	2	2	2	18

PRP Techniques

Bohlen et al. obtained 4-7 mL of PRP from 54 mL of patient blood [20], whereas Boden et al. obtained 3 mL of PRP from 30 mL of patient whole blood [21]. See Table 3 for more details on the PRP preparation process.

**Table 3:** PRP preparation process NR: not reported; PRP: platelet-rich plasma

Study	Volume	PRP Type	Buffering Agent	Device Used (Device Manufacturer)
Bohlen et al. [20]	4 - 7 mL	leukocyte-rich (LR-PRP)	ACD-A anticoagulant	Harvest SmartPrep Multicellular Processing System (Terumo BCT).
Boden et al. [21]	3 mL	leukocyte-poor (LP-PRP)	NR	Emcyte, Fort Myers, FL, USA

Pain Relief

In the included studies, the outcomes of PRP and surgical interventions were similar, with no significant statistical differences. In both studies, Bohlen et al. and Boden et al. reported an excellent outcome after the PRP and surgical interventions where the patient returned to full activity with no pain [20,21].

Outcome Measures

The Nirschl grading method was used by Bohlen et al., who discovered no statistically significant difference in success rates (P =0.37) between the two groups [20]. Regarding the visual analog scale (VAS), both groups demonstrated comparable scores (Surgery: 4.7 and PRP: 3.7; P= 0.12). In the surgical group, the mean Oxford Elbow Score (OES) and Mayo Elbow Performance Score (MEPS) scores were 42.2 and 93.5%, respectively, while in the PRP group, they were 45.9 and 92.3%, respectively. Both groups were comparable in terms of MEPS (P = 0.30) and OES (P = 0.18). Moreover, in terms of time to achieve pain-free status, a substantial difference was observed between both groups. Because the criteria for the desired outcome varies across studies and between anomalies, determining the efficacy of PRP for various musculoskeletal disorders can be difficult. The second aspect is that PRP use varies based on the ailment being treated.

Repeated-measures analyses using a means model utilizing the SAS MIXED procedure (SAS Institute Inc., Cary, NC) were employed by Boden et al. to provide independent estimates of the means by treatment group and time in the trial for quality-of-life, VAS pain, and Quick Disabilities of Arm, Shoulder & Hand (QDASH) scores (baseline and after the procedure) [21]. In repeated measurements, it was hypothesized that all outcomes followed a compound symmetrical variance-covariance form; using these assumptions, robust estimates of the standard error of parameters were utilized to conduct statistical tests and provide 95% confidence intervals for all outcomes. As long as the missing data are not informative and the model is accurate, model-based means remain unbiased even in the face of imbalanced and missing data (missing at random). Each model contained many predictor factors, such as treatment group, follow-up duration, and the interaction between the two. Within the mixed-effects linear model context, t-tests were utilized to compare model-based means and perform all other necessary statistical analyses. Results were summarised by calculating adjusted means and 95% confidence intervals for each treatment group and follow-up time point. All statistical tests were two-sided, and no effort was made to correct any bias introduced by making multiple comparisons. When the p-value was less than 0.05, they considered the result significant. In order to detect a difference in VAS score, they needed cohorts of 30 patients for each treatment group, according to the sample size calculations for a paired t-test using an alpha of 0.05 and an effect size of 0.50 performed before research started. Furthermore, a simple matched-pair t-test was conducted on the available and relevant data.

Discussion

The use of PRP in treating musculoskeletal issues has recently gained attention due to its promise as a promising option for patients who tried all non-surgical therapy options. Although there is a debate over the usefulness of PRP, studies have shown that it is a viable alternative to steroid injections and surgery in treating various illnesses and injuries. Thus, it remains a topic of constant investigation in the field of orthopedics to this day [12].

In the treatment of a variety of tendinopathies, including LE, PRP has been proven to be helpful. Using data from a randomized controlled trial (RCT), Peerbooms et al. reported the treatment of LE in 100 patients with PRP injections versus corticosteroid injections [13]. The study was conducted in the Netherlands and involved 100 participants. According to their findings, PRP performed substantially better than steroid injections, with favorable outcomes recorded in 73% of elbows treated with PRP. It was determined that the trial was a success when the VAS and DASH score improved by 25% one year after the study's beginning. Brkljac et al. discovered that 88.2% of patients who received PRP for intractable LE had favorable results, defined as an improvement on the Oxford Elbow Score (OES) [22]. In addition, it has been explored if PRP may be used to treat other types of tendinopathies besides Achilles tendinitis. In the Journal of Orthopaedic and Sports Physical Therapy, Vetrano et al. compared PRP with ECSWT in patients with patellar tendonitis [23]. Their findings showed that at each follow-up assessment, patients in both groups exhibited statistically significant symptom improvement. However, at the two-month follow-up, there were no statistically significant changes in the modified Blazina scale, VAS, or Victorian Institute of Sport Assessment-Patellar (VISA-P) scores across groups (P =0.339, 0.360, and 0.635), respectively. There was a statistically significant difference between the PRP and ESWT groups at 12 months, with the PRP group showing more improvement on the VISA-P, VAS, and modified Blazina scales. Fitzpatrick et al. highlighted that PRP was superior to corticosteroids in terms of recovery and outcomes, with 65.8% of patients experiencing a full recovery and successful outcomes following the administration of PRP [24].

Ongoing research into the use of PRP for the treatment of LE is being carried out on a large scale. In the meta-analysis of Arirachakaran and colleagues [25], autologous blood injections were associated with a higher risk of adverse effects than PRP injections, but not by a significant margin. According to the findings of that study, the risk of harmful effects from PRP injections was lower than the risk of adverse effects from autologous blood injections. It is comparable to the review conducted by Rodik and McDermott [17], which examined four studies comparing the effects of PRP injections with alternate injection treatments for LE and found that PRP injections were superior to alternate injection treatments in the treatment of LE. In the end, they discovered that, compared to whole blood or corticosteroid injections, PRP injections provided superior pain relief and improved functional outcomes 1 to 2 years after the injection was performed. Given the similarities between LE and multiple sclerosis (MS), these high-powered trials suggest that PRP injections may have a role in treating LE. While there is some evidence to support using PRP for treating ME, the evidence is lacking.

Although PRP injections have been studied extensively with different conditions, only a few studies have evaluated their efficacy in comparison with more invasive methods for treating ME. In a trial including 62 elbows, Boden et al. compared the Tenex method to PRP injections to treat ME and LE [21]. QuickDASH and VAS pain levels improved significantly in both groups; however, both groups were comparable. Eighty percent of Tenex patients and 79.3 percent of PRP patients expressed satisfaction with the intervention, which is not statistically different. They used the leukocyte-poor PRP (LP-PRP) rather than two injections of leukocyte-rich PRP (LR-PRP) as they believed that LP-PRP is associated with a reduced risk of a proinflammatory response after injection.

Despite a large body of research testing the efficacy of PRP in treating LE, there is no consensus on whether or not it should be used. In part, this is because various preparation procedures are employed, as well as a lack of standardization in the method of application used. Researchers from the University of Pennsylvania conducted an RCT to compare LP-PRP and LR-PRP. They discovered that there was no statistically significant difference between the two groups in terms of pain or function when compared to a control group that received saline solution. As a result, it is conceivable that the study did not sufficiently account for the effects of a time-dependent treatment because the trial's endpoints were at 4 and 8 weeks, respectively. We had a longer follow-up time of seven months for the PRP cohort, which may have influenced some results. According to Behera et al. [26], a study comparing the use of LP PRP with bupivacaine revealed that the use of LP PRP was related to time-dependent advantage in terms of pain scores and patient-reported outcomes at six months and one year when compared to a control group. The research team of Fitzpatrick et al. did a meta-analysis of numerous PRP preparation techniques and injection strategies and discovered that LR PRP techniques were much more effective than LP PRP techniques in improving wound healing [24].

Limitations

We acknowledge that our study has some limitations, including the limited coverage period (between 2010 and 2020) and the scarcity of studies dealing with ME, as most available studies dealt with LE. Additionally, the studies included in this review are of level III, and only studies written in English were covered; this may lead to publication bias.

## Conclusions

There is a lack of high-quality studies regarding the safety and efficacy of PRP in patients with ME. Evidence from the reviewed studies shows that PRP injections are just as effective as ME surgery in relieving pain and restoring function for those with ME, especially in the short and mid-term. Success rate, pain-free status, and quality of life were comparable in both the PRP and surgery groups. However, based on the number and quality of reviewed studies, these findings should be interpreted with caution. Further prospective multicenter studies with a larger sample size and adequate follow-up period are required to acquire more data regarding the safety, efficacy, and cost-effectiveness of PRP in ME.

## References

[1] Amin NH, Kumar NS, Schickendantz MS (2015). Medial epicondylitis: evaluation and management. J Am Acad Orthop Surg.

[2] Shiri R, Viikari-Juntura E, Varonen H, Heliövaara M (2006). Prevalence and determinants of lateral and medial epicondylitis: a population study. Am J Epidemiol.

[3] Descatha A, Leclerc A, Chastang JF, Roquelaure Y (2003). Medial epicondylitis in occupational settings: prevalence, incidence and associated risk factors. J Occup Environ Med.

[4] Wolf JM, Mountcastle S, Burks R, Sturdivant RX, Owens BD (2010). Epidemiology of lateral and medial epicondylitis in a military population. Mil Med.

[5] Morrey B (2014). Master Techniques in Orthopaedic Surgery: The Elbow, Third Edition. Shoulder Elbow.

[6] Otoshi K, Kikuchi S, Shishido H, Konno S (2014). The proximal origins of the flexor-pronator muscles and their role in the dynamic stabilization of the elbow joint: an anatomical study. Surg Radiol Anat.

[7] Tajika T, Oya N, Ichinose T (2020). Flexor pronator muscles' contribution to elbow joint valgus stability: ultrasonographic analysis in high school pitchers with and without symptoms. JSES Int.

[8] Vinod AV, Ross G (2015). An effective approach to diagnosis and surgical repair of refractory medial epicondylitis. J Shoulder Elbow Surg.

[9] Glousman RE, Barron J, Jobe FW, Perry J, Pink M (1992). An electromyographic analysis of the elbow in normal and injured pitchers with medial collateral ligament insufficiency. Am J Sports Med.

[10] Vangsness CT Jr, Jobe FW (1991). Surgical treatment of medial epicondylitis. Results in 35 elbows. J Bone Joint Surg Br.

[11] Ollivierre CO, Nirschl RP, Pettrone FA (1995). Resection and repair for medial tennis elbow. A prospective analysis. Am J Sports Med.

[12] Mlynarek RA, Kuhn AW, Bedi A (2016). Platelet-rich plasma (PRP) in orthopedic sports medicine. Am J Orthop (Belle Mead NJ).

[13] Peerbooms JC, Sluimer J, Bruijn DJ, Gosens T (2010). Positive effect of an autologous platelet concentrate in lateral epicondylitis in a double-blind randomized controlled trial: platelet-rich plasma versus corticosteroid injection with a 1-year follow-up. Am J Sports Med.

[14] Krogh TP, Fredberg U, Stengaard-Pedersen K, Christensen R, Jensen P, Ellingsen T (2013). Treatment of lateral epicondylitis with platelet-rich plasma, glucocorticoid, or saline: a randomized, double-blind, placebo-controlled trial. Am J Sports Med.

[15] Martínez-Montiel O, Valencia-Martinez G, Blanco-Bucio P, Villalobos-Campuzano C (2015). Treatment of elbow epicondylitis with platelet rich plasma versus local corticosteroids [Article in Spanish]. Acta Ortop Mex.

[16] Gautam VK, Verma S, Batra S, Bhatnagar N, Arora S (2015). Platelet-rich plasma versus corticosteroid injection for recalcitrant lateral epicondylitis: clinical and ultrasonographic evaluation. J Orthop Surg (Hong Kong).

[17] Rodik T, McDermott B (2016). Platelet-rich plasma compared with other common injection therapies in the treatment of chronic lateral epicondylitis. J Sport Rehabil.

[18] Muthu S, Patel S, Gobbur A, Patil SC, Ks KH, Yadav V, Jeyaraman M (2022). Platelet-rich plasma therapy ensures pain reduction in the management of lateral epicondylitis - a PRISMA-compliant network meta-analysis of randomized controlled trials. Expert Opin Biol Ther.

[19] Slim K, Nini E, Forestier D, Kwiatkowski F, Panis Y, Chipponi J (2003). Methodological index for non-randomized studies (MINORS): development and validation of a new instrument. ANZ J Surg.

[20] Bohlen HL, Schwartz ZE, Wu VJ, Thon SG, Finley ZJ, O'Brien MJ, Savoie FH 3rd (2020). Platelet-rich plasma is an equal alternative to surgery in the treatment of type 1 medial epicondylitis. Orthop J Sports Med.

[21] Boden AL, Scott MT, Dalwadi PP, Mautner K, Mason RA, Gottschalk MB (2019). Platelet-rich plasma versus Tenex in the treatment of medial and lateral epicondylitis. J Shoulder Elbow Surg.

[22] Brkljac M, Kumar S, Kalloo D, Hirehal K (2015). The effect of platelet-rich plasma injection on lateral epicondylitis following failed conservative management. J Orthop.

[23] Vetrano M, Castorina A, Vulpiani MC, Baldini R, Pavan A, Ferretti A (2013). Platelet-rich plasma versus focused shock waves in the treatment of jumper's knee in athletes. Am J Sports Med.

[24] Fitzpatrick J, Bulsara M, Zheng MH (2017). The effectiveness of platelet-rich plasma in the treatment of tendinopathy: a meta-analysis of randomized controlled clinical trials. Am J Sports Med.

[25] Arirachakaran A, Sukthuayat A, Sisayanarane T, Laoratanavoraphong S, Kanchanatawan W, Kongtharvonskul J (2016). Platelet-rich plasma versus autologous blood versus steroid injection in lateral epicondylitis: systematic review and network meta-analysis. J Orthop Traumatol.

[26] Behera P, Dhillon M, Aggarwal S, Marwaha N, Prakash M (2015). Leukocyte-poor platelet-rich plasma versus bupivacaine for recalcitrant lateral epicondylar tendinopathy. J Orthop Surg (Hong Kong).

